# Phosphoproteomics characterization of novel phosphorylated sites of lens proteins from normal and cataractous human eye lenses

**Published:** 2011-01-19

**Authors:** Chun-Hao Huang, Yi-Ting Wang, Chia-Feng Tsai, Yu-Ju Chen, Jiahn-Shing Lee, Shyh-Horng Chiou

**Affiliations:** 1 Graduate Institute of Medicine and Center for Research Resources and Development, Kaohsiung Medical University, Kaohsiung, Taiwan; 2 Institute of Biological Chemistry, Academia Sinica, Taipei, Taiwan; 3 Institute of Chemistry, Academia Sinica, Taipei, Taiwan; 4 Chemical Biology and Molecular Biophysics Program, Taiwan International Graduate Program, Institute of Chemistry, Academia Sinica, Taipei, Taiwan; 5 Institute of Biochemical Sciences, National Taiwan University, Taipei, Taiwan; 6 Department of Chemistry, National Taiwan University, Taipei, Taiwan; 7 Department of Ophthalmology, Chang-Gung Memorial Hospital, Chang-Gung University, Taipei, Taiwan

## Abstract

**Purpose:**

Post-translational modification (PTM) of lens proteins is believed to play various roles in age-related lens function and development. Among the different types of PTM, phosphorylation is most noteworthy to play a major role in the regulation of various biosignaling pathways in relation to metabolic processes and cellular functions. The present study reported the quantitative analysis of the in vivo phosphoproteomics profiles of human normal and cataractous lenses with the aim of identifying specific phosphorylation sites which may provide insights into the physiologic significance of phosphorylation in relation to cataract formation.

**Methods:**

To improve detection sensitivity of low abundant proteins, we first adopted SDS-gel electrophoresis fractionation of lens extracts to identify and compare the protein compositions between normal and cataractous lenses, followed by tryptic digestion, enrichment of phosphopeptides by immobilized metal affinity chromatography (IMAC) and nano-liquid chromatography coupled tandem mass spectrometry (nanoLC-MS/MS) analysis.

**Results:**

By comprehensively screening of the phosphoproteome in normal and cataractous lenses, we identified 32 phosphoproteins and 73 phosphorylated sites. The most abundantly phosphorylated proteins are two subunits of β-crystallin, i.e., βB1-crystallin (12%) and βB2-crystallin (12%). Moreover, serine was found to be the most abundantly phosphorylated residue (72%) in comparison with threonine (24%) and tyrosine (4%) in the lens phosphoproteome. The quantitative analysis revealed significant and distinct changes of 19 phosphoproteins corresponding to 28 phosphorylated sites between these two types of human lenses, including 20 newly discovered novel phosphorylation sites on lens proteins.

**Conclusions:**

The shotgun phosphoproteomics approach to characterize protein phosphorylation may be adapted and extended to the comprehensive analysis of other types of post-translational modification of lens proteins in vivo. The identification of these novel phosphorylation sites in lens proteins that showed differential expression in the cataractous lens may bear some unknown physiologic significance and provide insights into phosphorylation-related human eye diseases, which warrant further investigation in the future.

## Introduction

Human eye lenses are composed of elongated fiber cells, in which about 90% of total soluble proteins belong to three major classes of proteins, i.e., α-, β- and γ-crystallins [1,2]. Essentially these crystallins can exist in the eye lens with little turnover throughout the entire human lifespan albeit with various degrees of post-translational modification (PTM). Various types of PTM have been identified in animal eye lenses including especially human lenses, e.g.: 1. Deamidation [3,4], 2. Non-enzymatic glycosylation or glycation [5,6], 3. Oxidation of some amino acid residues of lens proteins such as tryptophan and methionine [7,8], 4. Sulfhydryl-disulfide oxidation [9,10], 5. Acetylation of NH_2_-terminal and lysine residues [11,12], 6. Truncation of crystallins [13,14], and 7. Phosphorylation [15-23]. Among these, phosphorylation is most noteworthy to play a major role in the regulation of various biosignaling pathways in relation to metabolic processes and cellular functions [24-26], which may include cancer development, aging, and cataract formation. Therefore, identification of protein phosphorylation and its exact phosphorylated residues in proteins or enzymes of interest are always considered as a preeminent and nontrivial task in the conventional structural and functional study of various cellular proteins. Mainly attributable to the recent advent and state-of-the-art instrumentation of proteomics, the investigation of protein phosphorylation has gradually become more amendable to routine analysis.

The recent explosion in available genomic sequence information is providing a useful sequence infrastructure for proteomics database. A major aspect of various proteomics strategies is the determination of protein identity (Protein ID) using analytical ‘‘fingerprints’’ or peptide mass fingerprinting (PMF) generated by digestion of proteins with specific enzymes such as trypsin, from which tandem mass (MS/MS) spectra of peptide fragments can then be used for comparison and confirmation of protein ID in available sequence databanks. The strategy based on the direct analysis of peptides generated from protein digestion by high-resolution liquid chromatographies coupled with tandem MS/MS spectrometry has facilitated the so-called “shotgun proteomics” for the identification of protein mixtures from any tissues of interest. Various MS/MS spectra can be algorithmically compared with predicted peptide spectra from sequence databases to identify the respective proteins. By combining with the recent development of capillary multidimensional liquid chromatography (capillary-MDLC), this shotgun proteomics approach is capable of characterizing proteins directly from entire cell lysates [27-31]. In shotgun proteomics, MDLC is a necessity to reduce sample complexity and increase dynamic range of protein identification. Recently mass spectrometric methods are being developed along the line that not only identifies proteins in a mixture but also compares the relative levels of protein expression between two different samples, i.e., quantitative shotgun proteomics.

The serious drawback of conventional gel-based 2-D gel proteomics lies in low sensitivity and under-representation for some special classes of proteins such as the extremely basic or acidic groups of proteins and membrane proteins [32-34]. In our previous study [35], phosphorylated peptides from trypsin-digested total protein mixtures of porcine lenses were concentrated and enriched on IMAC followed by identification of phosphopeptides on μLC-MS/MS. Gel-free IMAC phosphopeptide enrichment coupled with μLC-MS/MS analysis was found to be capable of identifying phosphorylated sites of various proteins from the whole lens extract. In this study, we have further applied quantitative shotgun proteomics to study and compare protein phosphorylation between normal and cataractous lens extracts to provide some basis to probe the role of phosphorylation in relation to cataract formation in vivo.

## Methods

### Materials and biologic tissues

Normal (30-year-old) and cataractous (68-year-old, Grade III of nuclear sclerosis) human lenses were obtained post mortem from the Department of Ophthalmology, Chang Gung Memorial Hospital, Taipei, Taiwan (J.-S. Lee). Eye lenses were kept and stored at −80 °C freezer before dissection. Triethylammonium bicarbonate (TEABC) and iron chloride (FeCl_3_) were purchased from Sigma Aldrich (St. Louis, MO). The BCA^TM^ protein-assay reagent kit was obtained from Pierce (Rockford, IL). Ammonium persulfate and N,N,N’,N’-tetramethylenediamine were purchased from Amersham Pharmacia (Piscataway, NJ). Reagent-grade acetic acid (AA) was purchased from J. T. Baker (Phillipsburg, NJ). Trifluoroacetic acid (TFA), formic acid (FA) and HPLC-grade acetonitrile were purchased from Sigma Aldrich. Chemically-modified and sequencing-grade trypsin was purchased from Promega (Madison, WI).

### Preparation of lens extracts

Lenses were homogenized and suspended in 20 mM Tris-HCl, pH 6.8 buffer containing 0.1% SDS and centrifuged for 30 min at 20,000× g for the extraction of total lens proteins as described previously [36-38].

### 1-D gel SDS–PAGE

After estimation of protein content by using a BCA^TM^ protein-assay reagent kit (Pierce, Rockford, IL), 10 μg of proteins in lens extracts were loaded on 12.5% one-dimensional SDS–PAGE for protein separation, followed by staining with Coomassie brilliant blue R-250 and destained in 10% methanol/ 7% acetic acid.

### In-gel digestion and nanoLC-ESI-MS/MS

Based on the SDS–PAGE analysis of samples, differentially expressed proteins were selected for further identification by nanoLC-MS/MS. The protein bands separated on 1-D SDS–PAGE were cut from gels, and then destained three times with 25 mM ammonium bicarbonate buffer (pH 8.0) in 50% acetonitrile (ACN) for 1 h. The gel pieces were dehydrated in 100% ACN for 5 min and then dried for 30 min in a vacuum centrifuge. Enzyme digestion was performed by adding 0.5 μg trypsin in 25 mM ammonium bicarbonate buffer per sample at 37 °C for 16 h. The peptide fragments were extracted twice with 50 μl 50% ACN/ 0.1% TFA. After removal of ACN and TFA by centrifugation in a vacuum centrifuge, samples were dissolved in 0.1% formic acid/ 50% ACN and analyzed by nanoLC-ESI-MS/MS at the core facility laboratory of the Center for Research Resources and Development, Kaohsiung Kaohsiung Medical University, Kaohsiung, Taiwan and at Institute of Chemistry, Academia Sinica, Taipei, Taiwan. Proteins were identified in the NCBI databases by use of MS/MS ion search with the search program MASCOT as described previously [35].

### Gel-assisted digestion

The protein samples from lenses were subjected to gel-assisted digestion. The sample was incorporated into a gel directly in an Eppendorf vial with acrylamide/bisacrylamide solution (40%, v/v, 29:1), 10% (w/v) APS, 100% TEMED in a proportion of 14:5:0.7:0.3. The gel was cut into small pieces and washed several times with 25 mM TEABC containing 50% (v/v) ACN. The gel samples were further dehydrated with 100% ACN and completely dried using a SpeedVac (ASAHI TECHNO GLASS Corp., Tokyo, Japan). Proteolytic digestion was then performed with trypsin (protein: trypsin=50:1, w/w [g/g]) in 25 mM TEABC with incubation overnight at 37 °C. The tryptic peptides were dried completely under vacuum and stored at −30 °C.

### IMAC preparation and protocol

This step of sample preparation and procedure is most critical for a successful phosphoproteomics study of complex protein mixtures isolated from biologic tissues. The IMAC column was first capped one end with a 0.5 μm frit disk enclosed in stainless steel column-end fitting. The Ni-nitrilotriacetic acid (Ni-NTA) resin was extracted from spin column (Qiagen, Hilden, Germany) and packed into a 10-cm microcolumn (500 μm i.d. PEEK column, Upchurch Scientific/Rheodyne, Oak Harbor, WA) as described previously [39]. Automatic purification of phosphopeptides was performed by connecting to an autosampler in an HP1100 solvent delivery system (Hewlett-Packard, Palo Alto, CA) with a flow rate 13 µl/min. First, the Ni^2+^ ions were removed with 100 µl 50 mM EDTA in 1 M NaCl. Then the IMAC column was activated with 100 µl 0.2 M FeCl_3_ and equilibrated with loading buffer for 30 min before sample loading. The loading buffer/ acetic acid was 6% (v/v) and the pH was adjusted to 3.0 with 0.1 M NaOH (pH=12.8). The peptide samples from trypsin digestion were reconstituted in the loading buffer and loaded into the IMAC column that had been equilibrated with the same loading buffer for 20 min. Then the unbound peptides were removed with 100 μl washing solution consisting of 75% (v/v) loading buffer and 25% (v/v) ACN, followed by equilibration with loading buffer for 15 min. Finally, the bound peptides were eluted with 100 µl 200 mM NH_4_H_2_PO_4_ (pH 4.4). Eluted peptide samples were dried under vacuum and then reconstituted in 0.1% (v/v) TFA (40 μl) for further desalting and concentration using ZipTips^TM^ (Millipore, Bedford, CA).

### NanoLC-MS/MS analysis

Purified phosphopeptide samples from about 500 µg total protein extract were reconstituted in 4 µl buffer A (0.1% formic acid (FA) in H_2_O) and analyzed by LC-Q-TOF MS (Waters Q-TOF^TM^ Premier; Waters Corp, Milford, MA). For LC-MS/MS analysis by Waters Q-TOF^TM^ Premier system, samples were injected into a 2 cm×180 μm capillary trap column and separated by 20 cm×75 μm Waters1 ACQUITYTM 1.7 mm BEH C18 column using a nanoACQUITY Ultra Performance LC^TM^ system (Waters Corp., Milford, MA). The column was maintained at 35 °C and bound peptides were eluted with a linear gradient of 0%–80% buffer B (buffer A, 0.1% FA in H_2_O; buffer B, 0.1% FA in ACN) for 120 min. MS was operated in ESI positive V mode with a resolving power of 10,000. NanoLockSpray source was used for accurate mass measurement and the lock mass channel was sampled every 30 s. The mass spectrometer was calibrated with a synthetic human [Glu^1^]-fibrinopeptide B solution (1 pmol/µl, from Sigma Aldrich) delivered through the NanoLockSpray source. Data acquisition was operated in the data directed analysis (DDA). The method included a full MS scan (m/z 400–1600, 0.6 s) and 3 MS/MS scans (m/z 100–1990, 1.2 s each scan) sequentially on the three most intense ions present in the full scan mass spectrum.

### Database search and data processing/filtering

Raw MS/MS data were converted into peak lists using Distiller (version 2.0; Matrix Science, London, UK) with default parameters. All MS/MS samples were analyzed using Mascot (version 2.2.1; Matrix Science). Mascot was set up to search the Swissprot_Mammalia (version 54.2, 55307 entries) assuming trypsin as the digestion enzyme. MASCOT was searched with a fragment ion mass tolerance of 0.1 Da and a parent ion tolerance of 0.1 Da. Two missed cleavages were allowed for trypsin digestion. Phosphorylation (Ser/Thr/Tyr) and oxidation (Met) were selected as two variable modifications. To evaluate the false discovery rate of protein identification, we repeated the search using identical search parameters and validation criteria against a randomized decoy database created by MASCOT. The false discovery rates with MASCOT score >36 (p<0.05) was 0.73% in our phosphoproteomics study of lens protein extracts.

### Label-free quantitation method

The quantitative analysis of peptides in the label-free experiments was performed by employing our recently published software, IDEAL-Q [40,41]. The raw data files acquired from Waters Q-TOF^TM^ Premier were converted into files of mzXML format by the program massWolf, and the search results in MASCOT were exported in eXtensive Markup Language data (.XML) format. After data conversion, the confident peptide identification results (p<0.05) from each LC-MS/MS run were loaded and merged to establish a global peptide information list (sequence, elution time, and mass-to-charge). Alignment of elution time is then performed based on the peptide information list using linear regression in different LC-MS/MS runs followed by correction of aberrational chromatographic shift across fragmental elution-time domains. To calculate relative peptide abundance, the tool performs reconstruction of extracted ion chromatography (XIC), and calculation of XIC area. The fold-change of a given peptide was calculated by the ratio of relative peptide abundance between different samples.

## Results and discussion

In spite of the biologic significance and physiologic role of protein phosphorylation and the rapid advances in MS methodologies, high-throughput characterization of site-specific phosphorylation residues in proteins is still challenged by the technical difficulties [42,43] associated with their dynamic modification patterns, substoichiometric concentrations, heterogeneous forms of phosphoproteins, and low sensitivity and response from MS analyses of total protein mixtures extracted from biologic tissues. Therefore improved methodologies that specifically enrich the transient phosphoproteome in a routine and comprehensive manner are important for studying phosphorylation-dependent cellular signaling associated with various diseased states [44].

### Experimental design and methodology evaluation

Identification of large numbers of phosphopeptides with high specificity, reproducibility and recovery is critical in phosphoproteomics analysis. IMAC takes advantage of the phosphate groups as electron donors that chelate metal ion (Fe^3+^-NTA-silica) to preferentially retain phosphopeptides. Although the simple and routinely used protocol yields adequate results for simple phosphoprotein mixtures, the results for proteome-wide analysis are far from satisfactory. As shown previously [35,39], we have found the IMAC protocol used herein can yield an efficient enrichment and obtain specific purification for phosphopeptides devoid of contamination (a lack of nonspecific competitive binding). The pH effect for the binding and elution of phosphopeptides in IMAC protocol has been critically evaluated, demonstrating that the current IMAC method can reflect the representative phosphorylated amino-acid distribution such as phosphotyrosine, phosphoserine and phosphothreonine in the cell without bias. To date, the specificity and recovery reported in our IMAC protocol significantly exceed those previously achieved by single-step IMAC or IMAC in combination with methylation [45]. This protocol demonstrated high specificity (98%) that was comparable with TiO_2_ chromatography [46,47]. As compared to two-step purification methods, our protocol provides comparable selectivity and low sample loss with some advantages over current procedures. In terms of practical use, it offers a simple one-step, more reproducible method amenable to automatic phosphopeptide purification and enrichment using a Fe^3+^-IMAC microcolumn. Greater than 90% column recovery and enrichment specificity can be routinely achieved for single IMAC purification of up to 1 mg of protein lysates from various cell lines or tissues.

### Gel-based 1D- or 2D-gel proteomics

In 1993, Henzel et al. [48] first reported and started the popular gel-based proteomics analysis by combining 2D-gel electrophoresis and mass spectrometry. The global identification of proteins in biologic samples was based on pre-separation of a protein mixture on 2-D gel electrophoresis. The mass spectral patterns from tandem mass (MS/MS) analysis of protein fragments generated from protease digestion were then compared with predicted peptide spectra from sequence databases to identify the respective proteins. Although previously 2-D gel electrophoresis coupled with tandem MS has been considered as the method of choice in proteomics study, only up to 2,000 individual polypeptide chains at most can be resolved on a single 2-D gel [31]. The number of detected proteins is still being relatively small as compared to the whole genome-encoded functional proteins of about 20,000~30,000 in higher vertebrates. It is especially under-representative of some special classes of proteins such as low-abundance transcription factors and membrane proteins [32-34] because of the low solubility of these classes of proteins in the first dimensional isoelectric-focusing (IEF) protein separation of 2-D gel electrophoresis in the absence of SDS denaturing agent. In our proteomic study of porcine lens proteins [35], we have also encountered poor solubility of some proteins in pre-MS 2-D gel separation.

**Figure 1:**
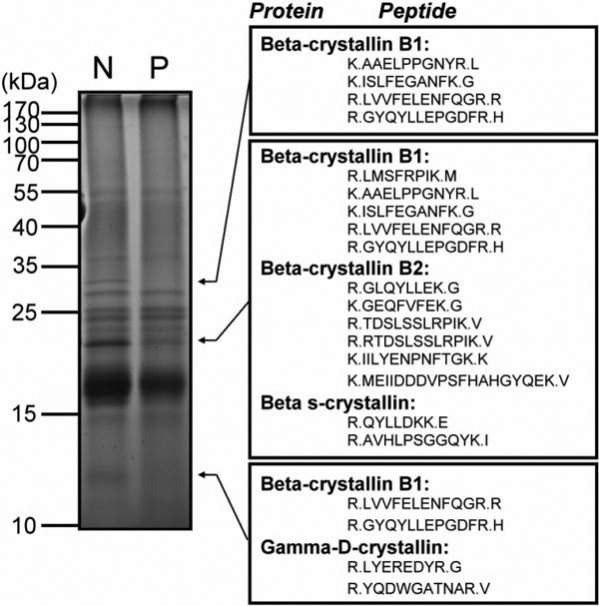
Comparative analysis of normal and cataractous human lens proteins by SDS–PAGE followed by LC-nanoESI-MS/MS. As shown in the left panel, a total of 10 μg lens proteins derived from normal (N) and cataractous (P) eye lenses were resolved with 12.5% SDS–PAGE and stained with Coomassie brilliant blue R-250. In the right panel, protein and peptide bands with different expression levels identified by LC-nanoESI-MS/MS were indicated by arrows. In comparison with normal human lens proteome, four crystallin proteins, β-crystallin B1, β-crystallin B2, βs-crystallin, and γD-crystallin in the cataract lens were found to significantly decrease in expression levels as compared to normal lens.

To improve the detection sensitivity for low abundant proteins, fractionation was first performed for the total protein extracts of normal and cataractous human lenses by 1-D SDS–PAGE gels (Figure 1). The proteins were separated into at least more than 10 different protein bands or zones from total protein mixtures of normal and cataract lenses. In comparison with normal human lens-proteome, only four crystallin proteins, i.e., β-crystallin B1, β-crystallin B2, βs-crystallin B1, and γD-crystallin in cataractous lens showed significant decrease in their expression levels. Similar results in previous reports also pointed to the important role of differential crystallin expression leading to cataract formation [49-51]. It can be seen that 1-D gels are less tedious and time-consuming than 2-D gels and still afford a respectable and extensive protein separation capable of protein ID analysis after LC-MS/MS. The unambiguous identification of some major classes of β- and γ-crystallin classes were confirmed and verified in addition to α-crystallins reported previously [35]. However, similar to the previous 2-D gel phosphoproteomic study of α-crystallins, the phosphorylated sites identified by 1-D gel-based methodology are still very limited; only a few well known abundant and predominant sites such as Ser-59, Ser-81 and Ser-155 in αA-crystallin, Ser-19, Ser-21 and Ser-59 in αB-crystallin, and Thr-189, Ser-9 and Ser-95 in βB1-crystallin were identified [35]. We could not find any other phosphopeptides in trypsin-digested protein bands corresponding to other lens proteins when using the 1-D or 2-D gel approach probably due to lower abundance of phosphopeptides generated from digestion of protein bands. Therefore we have resorted to the newer strategy of quantitative shotgun proteomics by using IMAC for the enrichment of phosphopeptides generated from the protease digestion of total lens extracts.

### Gel-free proteomic analysis of phosphorylated proteins in human lenses

**Figure 2:**
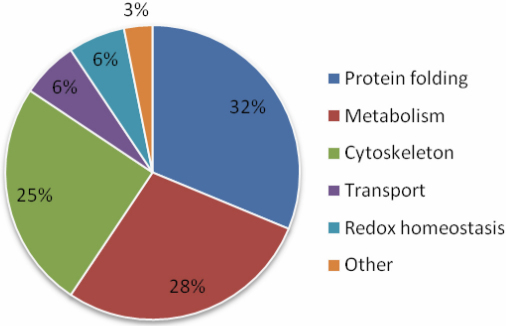
The percent distribution of annotated functions for identified phosphoproteins in normal human lenses. After being identified by using gel-free IMAC phosphopeptide enrichment and LC-MS/MS analysis, phosphoproteins were classified into five functional categories annotated in the proteomic databank. The proportions of annotated functions related to protein folding, metabolism, and cytoskeleton were 32%, 28%, and 25%, respectively. The other 15% identified proteins belonged to protein families of transport, cellular redox homeostasis, and other unidentified functions.

Because the capability of a gel-based proteomic approach to identify phosphoproteins was limited for phosphopeptide identification, we adopted instead a gel-free protocol similar to shotgun proteomic approaches [28,29]. By enrichment of the lens phosphopeptides on IMAC followed by LC-MS/MS analysis, we have identified 73 phosphorylation sites in human lens proteins (Table 1). As shown in Table 1, the identified 172 nondegenerate phosphopeptides belonged to 32 proteins in the human lens, including 9 crystallin proteins and other non-crystallin lens proteins possessing different cellular functions. Among the identified phosphoproteins, the relative proportions of the corresponding proteins with functions relating to protein folding, metabolism and cytoskeleton were 32%, 28%, and 25%, respectively (Figure 2). The other 15% phosphoproteins consisted of proteins with specified functions of transport, cellular redox system and homeostasis.

**Table 1:** Summary of identified phosphorylated proteins and phosphorylated sites in human lens proteins.

**Protein [Accession number]**	**Protein Mass, kDa**	**Fragment**	Phosphopeptides*	**Designation**
**Crystallin proteins**				
Alpha-crystallin B [P02511]	20.146	12–22	RPFFPFHSPSR	Ser-19
		12–22	RPFFPFHSPSR	Ser-21
		57–69	APSWFDTGLSEMR	Ser-59
		73–82	DRFSVNLDVK	Ser-76
		124–149	IPADVDPLTITSSLSSDGVLTVNGPR	Ser-139
Alpha-crystallin A [P02489]	19.897	13–21	TLGPFYPSR	Thr-13
		55–70	TVLDSGISEVRSDRDK	Ser-66
		79–88	HFSPEDLTVK	Ser-81
		146–157	IQTGLDATHAER	Thr-148
		146–157	IQTGLDATHAER	Thr-153
Beta-crystallin B1 [P53674]	28.006	25–50	GAPPAGTSPSPGTTLAPTTVPITSAK	Ser-32
		73–86	RAEFSGECSNLADR	Ser-77
		73–86	RAEFSGECSNLADR	Ser-81
		91–110	VRSIIVSAGPWVAFEQSNFR	Ser-93
		93–110	SIIVSAGPWVAFEQSNFR	Ser-97
		93–110	SIIVSAGPWVAFEQSNFR	Ser-107
		188–202	VSSGTWVGYQYPGYR	Ser-189
		188–202	VSSGTWVGYQYPGYR	Ser-190
		203–214	GYQYLLEPGDFR	Tyr-204
Beta-crystallin B2 [P43320]	23.365	90–101	RTDSLSSLRPIK	Thr-91
		90–101	RTDSLSSLRPIK	Ser-93
		91–101	TDSLSSLRPIK	Ser-95
		109–120	IILYENPNFTGK	Tyr-112
		109–120	IILYENPNFTGK	Thr-118
		146–160	VQSGTWVGYQYPGYR	Ser-148
		169–188	GDYKDSSDFGAPHPQVQSVR	Ser-174
		169–188	GDYKDSSDFGAPHPQVQSVR	Ser-175
Beta-crystallin A3 [P05813]	25.134	46–64	MEFTSSCPNVSERSFDNVR	Thr-49
		197–211	EWGSHAQTSQIQSIR	Ser-200
		197–212	EWGSHAQTSQIQSIRR	Ser-209
Beta-crystallin A4 [P53673]	22.360	49–71	VLSGAWVGFEHAGFQGQQYILER	Ser-51
		104–118	DSRLTIFEQENFLGK	Thr-108
Beta-crystallin S [P22914]	20.993	4–14	TGTKITFYEDK	Thr-6
		8–19	ITFYEDKNFQGR	Tyr-11
		85–95	AVHLPSGGQYK	Ser-90
		159–174	KPIDWGAASPAVQSFR	Ser-167
Gamma-crystallin B [P07316]	20.894	60–77	RGEYPDYQQWMGLSDSIR	Ser-75
Gamma-crystallin D [P07320]	20.725	16–32	HYECSSDHPNLQPYLSR	Ser-21
		61–77	GDYADHQQWMGLSDSVR	Ser-73
		61–77	GDYADHQQWMGLSDSVR	Ser-75
		153–163	RYQDWGATNAR	Thr-160
**Other proteins**				
Filensin [Q12934]	74.499	5–11	SYVFQTR	Ser-5
		230–239	EVLSHLQAQR	Ser-233
		452–467	VRSPKEPETPTELYTK	Ser-454
		454–467	SPKEPETPTELYTK	Thr-462
		457–467	EPETPTELYTK	Thr-460
		605–615	SRSLPEKGPPK	Ser-605
		607–615	SLPEKGPPK	Ser-607
Phakinin [Q13515]	45.851	32–43	SSSSLESPPASR	Ser-34
		77–89	ALGISSVFLQGLR	Ser-81
		77–89	ALGISSVFLQGLR	Ser-82
Fructose-bisphosphate aldolase C [P09972]	48.378	116–129	GILAADESVGSMAK	Ser-26
		130–144	RLSQIGVENTEENRR	Ser-132
Phosphoglycerate kinase 1 [P00558]	44.586	172–184	AHSSMVGVNLPQK	Ser-175
Heat shock protein beta-1 [P04792]	22.768	80–89	QLSSGVSEIR	Ser-82
Quinone oxidoreductase PIG3 [Q53FA7]	35.514	258–267	RGSLITSLLR	Ser-260
Aquaporin-5 [P55064]	41.943	395–403	KKTMELTTR	Thr-397
Peroxiredoxin-2 [P32119]	21.878	110–119	RLSEDYGVLK	Ser-112
Malate dehydrogenase [P40925]	19.897	239–248	KLSSAMSAAK	Ser-241
Pyruvate kinase isozymes M1/M2 [P14618]	58.025	247–255	KASDVHEVR	Ser-249
Coronin-1B [Q9BR76]	54.200	207–214	RGTLVAER	Thr-209
Actin-related protein 2/3 complex subunit 2 [O15144]	22.724	135–148	RASHTAPQVLFSHR	Thr-139
Plectin-1 [Q15149]	531.466	3783–3793	RLTAEDLFEAR	Thr-3785
Limbic system-associated membrane protein [Q13449]	37.370	89–97	RHSLEYSLR	Ser-91
Drebrin [Q16643]	71.385	140–147	LSSPVLHR	Ser-142
Ras-related C3 botulinum toxin substrate 2 [P15153]	21.415	164–174	GLKTVFDEAIR	Thr-167
Band 4.1-like protein 2 [O43491]	112.519	400–412	RLSMYGVDLHHAK	Ser-402
Nucleoside diphosphate kinase A [P15531]	17.138	89–105	VMLGETNPADSKPGTIR	Thr-94
Retinal dehydrogenase 1 [P00352]	54.827	411–419	FKSLDDVIK	Ser-413
Fructose-bisphosphate aldolase A [P04075]	39.395	29–43	GILAADESTGSIAKR	Ser-39
Glyceraldehyde-3-phosphate dehydrogenase [P04406]	36.030	163–186	VIHDNFGIVEGLMTTVHAITATQK	Thr-184
Lens fiber major intrinsic protein [P30301]	28.104	229–238	SISERLSVLK	Ser-235
Uncharacterized protein C3orf72 [Q6ZUU3]	18.613	32–40	LSESPALVK	Ser-35

The lens proteins analyzed herein were from normal (30-year-old) human lenses obtained post mortem from Department of Ophthalmology, Chang Gung Memorial Hospital (J.-S. Lee). This table shows phosphoproteins and their phosphorylated sites in normal human lenses identified by immobilized metal affinity chromatography (IMAC) and LC-MS/MS analysis. *Phosphorylation sites are underlined.

**Figure 3:**
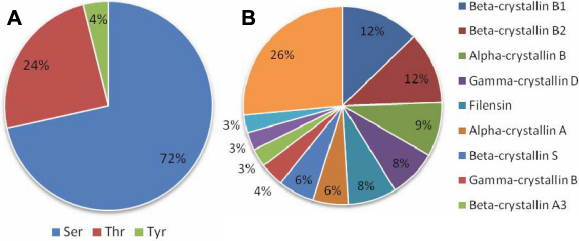
The percent distribution of phosphorylated sites in normal human lens proteins identified by using gel-free IMAC phosphopeptide enrichment and LC-MS/MS analysis. **A**: Proportions of three different phosphorylated amino-acid residues (Ser/Thr/Tyr) in normal human lens extract. Phosphorylation on serine (72%) was more common than threonine (24%) and tyrosine (4%). **B**: Proportions of the identified proteins with phosphorylation in normal human lens proteins. The proportions of phosphopeptides identified in βB1-crystallin, βB2-crystallin, αB-crystallin, γD-crystallin, filensin, αA-crystallin, and β-crystallin S (or denoted as βs-crystallin) were 12%, 12%, 9%, 8%, 8%, 6%, and 6%, respectively.

As shown in Figure 3A, further analysis of the whole phosphoproteome in human lenses indicated that phosphorylation on serine (72%) was more common than that on threonine (24%) and tyrosine (4%). In Figure 3B, most phosphopeptides were identified as being crystallin proteins, indicating that the major classes of lens crystallins are also the most abundant phosphoproteins in the human lens tissue. The proportions of phosphopeptides identified as being βB1-crystallin, βB2-crystallin, αB-crystallin, γD-crystallin, filensin, αA-crystallin, and βs-crystallin were 12%, 12%, 9%, 8%, 8%, 6%, and 6%, respectively, emphasizing the fact that βB-crystallin subunits are indeed the major phosphorylation targets in the lens and may play a significant role in the phosphorylation-related biosignaling function in this transparent lens tissue.

### Identification of phosphorylation sites in human lens crystallins

**Figure 4:**
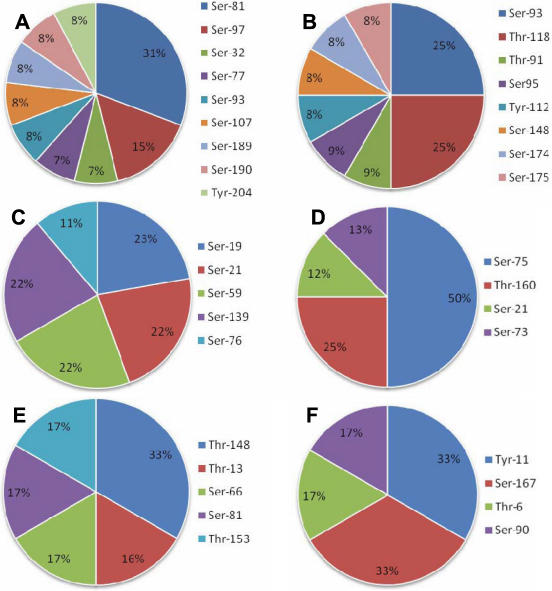
The percent distribution of phosphorylation sites of phosphorylated crystallin proteins in normal human lens proteins. Distribution of in vivo phosphorylation sites in **A**: βB1-crystallin; **B**: βB2-crystallin; **C**: αB-crystallin; **D**: γD -crystallin; **E**: αA-crystallin; and **F**: βS-crystallin. Ser-81 (31%) and Ser93/Thr-118 (25%) are the predominant phosphorylation-sites in βB1-crystallin and βB2-crystallin, respectively. In addition, the phosphorylation of αB-crystallin was shown to distribute almost evenly over the whole crystallin molecule at Ser-19 (23%), Ser-21 (22%), Ser-59 (22%), and Ser-139 (22%). In contrast, there was at least one predominant phosphorylated site in other crystallin proteins, i.e., Ser-75 (50%) in γD-crystallin, Thr-148 (33%) in αA-crystallin, and Tyr-11/Ser-167 (33%) in βS-crystallin.

As shown in Table 1, the phosphorylation sites of crystallin proteins were found to spread over the entire polypeptide regions of these crystallins. Based on the proportion of phosphorylation sites in each crystallin, we found that Ser-81 (31%) and Ser93/Thr-118 (25%) are the predominant phosphorylation-sites in βB1- and βB2-crystallin, respectively (Figure 4A,B). In addition, the phosphorylation of αB-crystallin was shown to distribute evenly over the whole crystallin molecule at Ser-19 (23%), Ser-21 (22%), Ser-59 (22%), and Ser-139 (22%; Figure 4C) similar to our previous report on porcine αB-crystallin [35]. In contrast, some predominant phosphorylation sites present in other crystallin proteins were also identified, e.g., Ser-75 (50%) in γD-crystallin, Thr-148 (33%) in αA-crystallin, and Tyr-11/Ser-167 (33%) in β-crystallin S (also denoted as βs-crystallin). The mechanisms underlying the differential phosphorylation at specific sites of these crystallins remain unknown, which should be of interest for further study in the future.

### Identification of phosphorylation sites in non-crystallin proteins involved in cytoskeleton, metabolism, transport, and cellular redox homeostasis

In addition to 9 lens crystallins, 23 non-crystallin proteins were also found to be phosphorylated in vivo in our phosphoproteomic analysis (Table 1). It is noteworthy that similar to αB-crystallin (a member of the small heat-shock protein family in the lens), another heat-shock protein beta-1 (homolog of heat-shock proteins Hsp27 and Hsp20) with chaperone activity was shown to be phosphorylated at Ser-82 [52,53]. To date these phosphorylated sites in the non-crystallin proteins have never been identified in the lens tissue and warranted for detailed functional characterization in the future.

### Comparative analysis of phosphoproteome in human lenses with or without cataract

To investigate the differential post-translational modification of human lens proteins with or without cataract, we performed quantitative phosphoproteomic analysis. As shown in Table 2, 19 phosphoproteins consisting of 8 crystallin proteins and 11 non-crystallin proteins with their corresponding 28 phosphorylated sites were identified between these two types of human lenses. Among these identified proteins, the extents of 15 phosphorylated sites were found to increase by twofold while 13 sites decreased in phosphorylation, indicating that complicated post-translational modification such as phosphorylation may be one of the causative factors underlying the development of human cataract. Furthermore, some quantitative changes in the phosphorylation status were found even in the same proteins from normal and diseased lenses such as βB1-crystallin and αB-crystallin. In human cataractous lenses, two phosphorylated sites in βB1-crystallin, Ser-32 and Ser-81, were found to decrease while Ser-93 increased in their relative phosphorylation ratios (P/N ratio in Table 2). The different proportions of phosphorylation and specific phosphorylated sites associated with normal and cataractous human lens proteins may form a firm basis for unraveling the mechanistic pathways of cataract formation with aging. Furthermore, among the differentially expressed phosphopeptides, 20 phosphorylation sites were verified to be newly discovered based on comparison with those in the phosphoprotein databases, Uni-Prot and PhosphoSitePlus* website. The data also revealed 14 novel phosphorylation sites on 7 crystallin proteins. These differentially expressed phosphorylation and their associated phosphorylated sites might be the potential therapeutic targets of cataract disease, which warrant further investigation.

**Table 2:** Summary of phosphorylated sites from normal and cataractous lenses.

**Protein [accession number]**	**Novel site**	**Designation**	**Fragment**	Peptide*	**m/z**	**Charge**	**Score**	**P/N ratio¶**
**Crystallin proteins**								
Beta-crystallin B1 [P53674]	Yes	Ser-32	25–50	GAPPAGTSPSPGTTLAPTTVPITSAK	1229.136	2	94.99	0.32
	Yes	Ser-81	73–86	RAEFSGECSNLADR	817.847	2	89.04	0.40
				RAEFSGECSNLADR	545.564	3	69.03	0.46
	Yes	Ser-93	91–110	VRSIIVSAGPWVAFEQSNFR	781.732	3	62.53	5.52
Beta-crystallin B2 [P43320]		Thr-91	90–101	RTDSLSSLRPIK	484.894	3	44.22	1.66
	Yes	Ser-148	146–160	VQSGTWVGYQYPGYR	920.923	2	110.26	2.69
	Yes	Ser-174	169–188	GDYKDSSDFGAPHPQVQSVR	757.332	3	64.55	2.49
	Yes	Ser-175	169–188	GDYKDSSDFGAPHPQVQSVR	757.345	3	66.7	2.30
Alpha-crystallin B [P02511]	No	Ser-19	12–22	RPFFPFHSPSR	485.557	3	68.97	1.89
	No	Ser-76	73–82	DRFSVNLDVK	636.793	2	66.09	2.09
Gamma-crystallin D [P07320]	Yes	Ser-21	16–32	HYECSSDHPNLQPYLSR	709.31	3	49.79	0.29
	Yes	Ser-73	61–77	GDYADHQQWMGLSDSVR	1022.934	2	57.89	0.23
	Yes	Ser-75	60–77	RGDYADHQQWMGLSDSVR	734.304	3	64.34	0.47
			61–77	GDYADHQQWMGLSDSVR	1022.929	2	79.19	0.21
Alpha-crystallin A [P02489]	Yes	Thr-153	146–157	IQTGLDATHAER	696.33	2	70.14	4.26
Beta-crystallin S [P22914]	Yes	Thr-6	4–14	TGTKITFYEDK	691.825	2	46.45	0.44
	Yes	Tyr-11	8–19	ITFYEDKNFQGR	799.348	2	74.57	0.48
Gamma-crystallin B [P07316]	Yes	Ser-75	60–77	RGEYPDYQQWMGLSDSIR	1141.009	2	74.11	0.11
Beta-crystallin A3 [P05813]	Yes	Ser-209	197–212	EWGSHAQTSQIQSIRR	655.3	3	58.65	5.41
**Other proteins**								
Phosphoglycerate kinase 1 [P00558]	Yes	Ser-175	172–184	AHSSMVGVNLPQK	732.304	2	48.65	10.70
Uncharacterized protein C3orf72 [Q6ZUU3]	Yes	Ser-35	32–40	LSESPALVK	512.237	2	39.15	4.84
Quinone oxidoreductase PIG3 [Q53FA7]	No	Ser-260	258–267	RGSLITSLLR	598.324	2	50.37	4.29
Aquaporin-5 [P55064]	Yes	Thr-397	395–403	KKTMELTTR	594.28	2	47.93	3.48
Peroxiredoxin-2 [P32119]	No	Ser-112	110–119	RLSEDYGVLK	630.302	2	40.74	3.18
Malate dehydrogenase [P40925]	No	Ser-241	239–248	KLSSAMSAAK	545.263	2	43.74	2.46
Pyruvate kinase isozymes M1/M2 [P14618]	Yes	Ser-249	247–255	KASDVHEVR	560.771	2	51.31	2.12
Phakinin [Q13515]	Yes	Ser-34	32–43	SSSSLESPPASR	642.748	2	52.18	0.46
Filensin [Q12934]	Yes	Ser-454	452–467	VRSPKEPETPTELYTK	652.331	3	64.65	0.35
Glyceraldehyde-3-phosphate dehydrogenase [P04406]	No	Thr-184	163–186	VIHDNFGIVEGLMTTVHAITATQK	897.788	3	58.72	0.29
Heat shock protein beta-1 [P04792]	No	Ser-82	80–89	QLSSGVSEIR	578.283	2	43.24	0.15

The lens proteins analyzed herein were from normal (30-year-old) and cataractous (68-year-old, Grade III of nuclear sclerosis) human lenses obtained post mortem from Department of Ophthalmology, Chang Gung Memorial Hospital (J.-S. Lee). This table shows the ratio of identified phosphorylated peptides in cataractous and normal human lenses by quantitative phosphoproteomic analysis. *Phosphorylation sites are underlined. ¶P, cataract patients; N, normal without cataract.

### Conclusions

The conventional gel-based phosphoproteomics analyses by 1-D SDS–PAGE coupled LC-MS/MS and separately by IMAC enrichment of phosphopeptides followed by shotgun label-free quantitation method have been used to analyze and compare phosphorylation patterns of lens proteins from whole tissue extracts of normal and cataractous lenses. In this report we have focused on employing efficient IMAC protocol of phosphopeptide enrichment for profiling and quantitative analysis of transiently phosphorylated proteins. The IMAC protocol reported herein demonstrated enrichment with high specificity and low sample loss without the need for additional esterification and desalting step. This procedure may be applicable to a variety of materials such as tissue, cell and body fluid. As judged by the higher sample recovery and greater number of phosphopeptides identified by the critically validated IMAC procedure [35,39] in this study as compared to previous reports on phosphorylation analysis in the literature [4,15-18,54,55], it should prove feasible for the routine phosphoproteome analysis in the future. The combination of this protocol with either stable isotope tagging or a label-free methodology may be further employed for large-scale comparative proteomic studies to decipher the dynamic and complicated phosphoproteomes from various biologic samples of diverse tissues. On the other hand, the identification of these novel phosphorylation sites in lens proteins that showed differential expression in the cataractous lens may bear some as-yet-unknown physiologic significance and provide insights into phosphorylation-related human eye diseases.
